# The Diagnosis and Management of Hyperinsulinaemic Hypoglycaemia

**DOI:** 10.4274/jcrpe.1891

**Published:** 2015-06-03

**Authors:** Klára Roženková, Maria Güemes, Pratik Shah, Khalid Hussain

**Affiliations:** 1 Charles University in Prague and University Motol Hospital, Department of Paediatrics, Prague, Czech Republic; 2 University College London, Institute of Child Health, Genetics and Epigenetics in Health and Disease, Genetics and Genomic Medicine Programme, London, UK; 3 Great Ormond Street Hospital for Children NHS Trust, Department of Paediatric Endocrinology, London, UK

**Keywords:** congenital hyperinsulinism, hypoglycaemia, 18Fluorine L-dihydroxyphenylalanine positron emission tomography scan, diazoxide, pancreatectomy, sirolimus

## Abstract

Insulin secretion from pancreatic β-cells is tightly regulated to keep fasting blood glucose concentrations within the normal range (3.5-5.5 mmol/L). Hyperinsulinaemic hypoglycaemia (HH) is a heterozygous condition in which insulin secretion becomes unregulated and its production persists despite low blood glucose levels. It is the most common cause of severe and persistent hypoglycaemia in neonates and children. The most severe and permanent forms are due to congenital hyperinsulinism (CHI). Recent advances in genetics have linked CHI to mutations in 9 genes that play a key role in regulating insulin secretion (ABCC8, KCNJ11, GLUD1, GCK, HADH, SLC16A1, UCP2, HNF4A and HNF1A). Histologically, CHI can be divided into 3 types; diffuse, focal and atypical. Given the biochemical nature of HH (non-ketotic), a delay in the diagnosis and management can result in irreversible brain damage. Therefore, it is essential to diagnose and treat HH promptly. Advances in molecular genetics, imaging methods (18F-DOPA PET-CT), medical therapy and surgical approach (laparoscopic surgery) have completely changed the management and improved the outcome of these children. This review provides an overview of the genetic and molecular mechanisms leading to development of HH in children. The article summarizes the current diagnostic methods and management strategies for the different types of CHI.

## INTRODUCTION

Hyperinsulinaemic hypoglycaemia (HH), one of the most frequent causes of persistent hypoglycaemia in neonates and infants, is a heterogeneous condition caused by dysregulation of insulin secretion from pancreatic β-cells. It is extremely vital to make a rapid diagnosis of HH and institute immediate appropriate management in these patients to prevent hypoglycaemic-related brain injury resulting in neurological complications like cerebral palsy, epilepsy and even death (1). The increased risk of brain injury in HH is due to the metabolic actions of insulin driving glucose into the insulin-sensitive tissues (skeletal muscle and adipose tissue) and inhibiting the glucose production via glycolysis and gluconeogenesis. Insulin also inhibits fatty acid release and ketone body synthesis; hence, the brain is deprived of both its primary and secondary energy sources (glucose and ketone bodies) (2). In addition to ketone bodies, lactate also provides an alternative cerebral fuel in hypoglycaemic newborns (3).

The most severe forms of HH are inherited and the term congenital hyperinsulinism (CHI) refers to these inherited forms of HH. However, HH can also be secondary to various risk factors like perinatal asphyxia, maternal diabetes mellitus, intra-uterine growth restriction (4), or associated with syndromes affecting growth like Beckwith-Wiedemann syndrome (BWS) and Sotos syndrome (5).

HH typically presents as fasting hypoglycaemia, but in some cases, the hypoglycaemia is provoked by protein/leucine loading or even exercise. Insulinoma, although a rare cause, should be considered in older children and adolescents presenting with recurrent hypoglycaemia. The clinical presentation is heterogeneous, patients can be completely asymptomatic, have a pharmacologically responsive mild disease or a severe disease un-responsive to medication needing surgical intervention (6).

**Figure 1:**
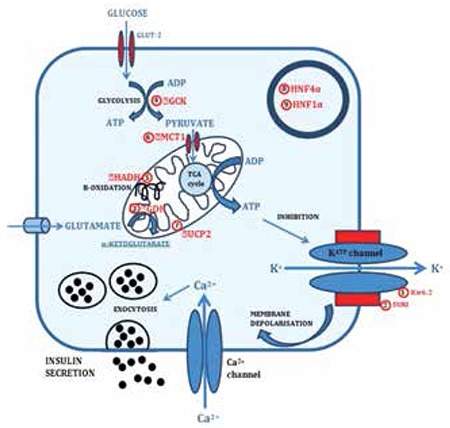
Genetic defects associated with CHI: (1) SUR1 subunit of the KATP channel encoded by ABCC8 gene; (2) Kir6.2 subunit of the KATP channel encoded by KCNJ11 gene; (3) Glutamate dehydrogenase (GDH) encoded by GLUD1 gene; (4) Glucokinase (GCK) encoded by GCK gene; (5) L-3-hydroxyacyl-coenzyme A dehydrogenase (HADH) encoded by HADH gene; (6) Monocarboxylate transporter (MCT1) encoded by SLC16A1 gene; (7) Uncoupling protein 2 (UCP2) encoded by UCP2 gene; (8) Hepatocyte nuclear factor 4α (HNF4α) encoded by HNF4A gene; (9) Hepatocyte nuclear factor 1α (HNF1α) encoded by HNF1A gene

CHI occurs due to mutations in key genes which play a role in insulin secretion from pancreatic β-cells. Currently mutations have been identified in nine different genes (ABCC8, KCNJ11, GLUD1, GCK, HADH, SLC16A1, UCP2, HNF4A and HNF1A) that lead to dysregulated secretion of insulin (5,7,8). The most common cause for CHI are mutations in the genes ABCC8 and KCNJ11 (both autosomal recessive and dominant) that encode the SUR1 and Kir6.2 subunits of the pancreatic β-cell KATP channel, respectively (9,10,11,12,13,14). The genetic defects associated with CHI are summarized in Figure 1.

Histologically, CHI is classified into three subgroups: diffuse, focal and atypical forms (15,16). In the diffuse disease, there are hyper-functioning pancreatic β-cells affecting the whole pancreas. Children with diffuse form of CHI due to recessive mutations in ABCC8 and KCNJ11 usually do not respond to diazoxide. Focal forms are sporadic in inheritance and associated with a paternally inherited mutation in ABCC8/KCNJ11 genes (17,18,19). Focal lesion may occur in any part of the pancreas, although the tail and body are the most common locations (20,21). Histologically, focal forms are characterised by nodular hyperplasia of islet cell and ductuloinsular complexes surrounded by histologically and functionally normal pancreatic tissue (22). In the “atypical” forms, there is an enlargement of pancreatic β-cell nuclei that is confined to discrete regions of the pancreas (23). This histological picture raises the possibility of mosaicism (24,25). An ABCC8 mutation and mosaic uniparental disomy has been reported to cause atypical form of CHI (25).

## Causes of HH

### Transient HH

Transient HH spontaneously resolves within a few days to up to a few months (26). It is usually secondary to maternal diabetes mellitus, intra-uterine growth restriction, perinatal asphyxia, polycythaemia, maternal administration of drugs such as sulphonylureas and intravenous glucose infusions during labour (9).

### Congenital Hyperinsulinism (CHI)

#### (a) Pancreatic β-cell KATP channel defects

Mutations in genes ABCC8 (SUR1 subunit) and KCNJ11 (Kir6.2 subunit) are the most common cause of CHI. Both the ABCC8/KCNJ11 genes are localized on chromosome 11p15.1. The most severe forms of CHI are due to recessive inactivating (loss of function) mutations in ABCC8 and KCNJ11 leading to unregulated insulin secretion despite severe hypoglycaemia (10,11). Dominant inactivating mutations in ABCC8 and KCNJ11 usually cause a milder form of CHI which is responsive to diazoxide (17,27). However, medically unresponsive forms have also been reported (28).

#### (b) Hyperinsulinism/hyperammonaemia syndrome (HI/HA)

Dominant missense mutations in the gene GLUD1, which encodes mitochondrial enzyme glutamate dehydrogenase (GDH) (29), cause HI/HA. As a result, the GDH activity is increased and that leads to inappropriate insulin secretion from pancreatic β-cells and excessive ammonia production. However, there is a rare group of patients who demonstrate leucine hypersensitivity but have a persistently normal serum ammonia level (15,30). Children usually have milder symptoms compared to other forms of CHI (15,30). Urinary α-ketoglutarate excretion is raised in HI/HA patients (31).

#### (c) Glucokinase gene mutations

Glucose is phosphorylated to glucose-6-phosphate by the enzyme glucokinase (GCK) that helps to control glucose metabolism in the β-cell (32). Mutations in GCK lead to increased ATP: ADP ratio in the pancreatic β-cell. This then results in KATP channel closure and inappropriate secretion of insulin (33). The activating GCK mutations are inherited in an autosomal dominant manner with varying severity of symptoms within the families and generally respond to diazoxide (34,35).

#### (d) HADH gene mutations

Mitochondrial L-3-hydroxyacyl-coenzyme A dehydrogenase (HADH) gene on chromosome 4q22-26 (36) encodes the enzyme HADH. Mutations in this gene are a rare cause of CHI (37). This enzyme activity is high in the pancreatic β-cells, where it converts L3-hydroxyacyl CoA to 3-ketoacyl CoA and then subsequently to 3-hydroxybutyryl-CoA, the penultimate step of the β-oxidation pathway. HADH gene mutations can lead either to severe neonatal CHI or to mild late-onset CHI (38). Some but not all patients with HADH mutations have abnormal organic acid profiles and acylcarnitines (15,30). So far, there has been no report of diazoxide-unresponsive hyperinsulinism in this group (39).

#### (e) Exercise-induced hyperinsulinism (SLC16A1)

SLC16A1 gene encodes monocarboxylate transporter 1 (MCT1) which is required for transport of pyruvate and lactate into pancreatic β-cells. Activating mutations in SLC16A1 increase the expression of MCT1 in the β-cells, which under normal conditions is very low. Strenuous exercise then leads to accumulation of lactate and pyruvate which results in pyruvate-stimulated insulin secretion (40). Medical treatment is usually not required and hypoglycaemic episodes can be prevented by avoiding strenuous exercise (41,42).

#### (f) UCP2 gene mutations

Uncoupling protein 2 (UCP2) disengages oxidative metabolism from ATP synthesis in the pancreatic β-cell. Mutations in UCP2 gene increase ATP synthesis and glucose-sensitive insulin secretion leading to HH (43).

#### (g) HNF4A and HNF1A gene mutations

Hepatocyte nuclear factor 4α (HNF4A) gene encodes for the transcription factor HNF4α, a nuclear hormone receptor involved in glucose-stimulated insulin secretion (44). Heterozygous mutations in the HNF4A gene have been reported to cause transient CHI in the newborn period (45,46) and maturity-onset diabetes of the young type 1 due to progressive β-cell dysfunction (47) in adolescence or early adulthood. Affected patients are usually born large for gestational age (LGA) and present with HH within the first week of life, which is responsive to diazoxide (48).

Recently, missense mutations in the HNF1A gene have been described to cause transient CHI. The clinical definition is not complete, but the phenotype is similar to the HNF4A mutations and these children may well be responsive to diazoxide (49,50).

### Postprandial forms of HH

Postprandial HH (PPHH) is a condition that causes hypoglycaemia within a few hours of meal ingestion due to inappropriate insulin secretion in response to the meal. “Dumping syndrome” is a type of PPHH in children who have undergone Nissen’s fundoplication/gastric bypass surgery (51,52). PPHH after Nissen’s fundoplication has an abnormally excessive secretion of glucagon-like peptide-1 (GLP-1) which may lead to exaggerated insulin secretion resulting in hypoglycaemia (53).

PPHH can also be caused by an autoimmune condition in which there is presence of insulin-binding autoantibodies in children who have not been previously exposed to exogenous insulin (54). PPHH has also been reported in some cases of insulin receptor gene mutations (leprechaunism) (55,56).

### Other causes of HH

Insulinoma, a rare form of neuroendocrine tumor, must be considered in older children or adolescents presenting with HH (57). Family history is extremely important as a diagnostic clue in familial cases where insulinoma may be a part of multiple endocrine neoplasia syndrome type 1.

## Clinical Presentation of HH

HH most commonly presents during the neonatal period. Although less frequently, HH can also manifest later in infancy, childhood or exceptionally in adolescence or adulthood, but usually with a milder phenotype.

The clinical symptoms of hypoglycaemia are nonspecific in the neonatal period and can include poor feeding, hypothermia, irritability, lethargy, apnoea, seizures and even coma. Symptoms generally develop after a period of fasting or when the child is unwell. In some forms of HH, the episodes of hypoglycaemia can be triggered by protein-rich meals (in HI/HA syndrome (and in HADH gene mutations), exertion [exercise-induced HI (EIHI)] (26) or postprandially [in dumping syndrome and insulin receptor gene mutations (58)].

Many developmental syndromes may be associated with HH (59). BWS is the most commonly associated as 50% of these children will present with HH (60). In the majority of BWS, HH will be transient to a few days, but in 5% of them, it will be permanent requiring medical therapy and in some cases - subtotal pancreatectomy (60). Other syndromes where HH has been described are those with: overgrowth [Sotos (61), Simpson-Golabi-Behmel (62)], growth failure [Kabuki (63), Costello (64)], chromosomal abnormalities [trisomy 13 (65), mosaic Turner (66), insulin receptor mutation (leprechaunism) (67,68) and others congenital disorders of glycosylation (69,70,71), congenital central hypoventilation syndrome] (72).

## Diagnosis of HH

The history should focus on identifying risk factors for HH; these include the presence of: maternal diabetes mellitus (pre and gestational), intra-partum drugs administered to the mother (glucose infusions, oral hypoglycaemic agents), eventful delivery (foetal distress, birth asphyxia), prematurity, small for gestational age (SGA) and LGA. When taking the family history, it is important to identify other family members with episodes of hypoglycaemia - possibly misdiagnosed as infantile seizures or unexplained deaths (58), but also diabetes mellitus and its onset age and the existence of consanguinity.

In the physical examination, special attention should be paid to auxology (macrosomia and SGA are more common in transient HH) and syndromic features to rule out developmental syndromes. BWS will typically manifest with macroglossia, hemihypertrophy, anterior abdominal wall defects, organomegaly, ear lobe creases, helical pits and renal tract abnormalities. The presence of hepatomegaly in patients with HH indicates the excessive glycogen deposition, but may also redirect the diagnosis towards metabolic conditions (e.g. glycogen storage disorders) and findings such as midline brain abnormalities, hyperpigmentation, undescended testes or micropenis may suggest hypopituitarism.

It is paramount to diagnose HH as early as possible to avoid hypoglycaemic brain injury. Despite the difficulty in defining a cut-off concentration of blood glucose that suits all ages and conditions that present with hypoglycaemia, the level most consistently used worldwide to define hypoglycaemia for patients with HH is 3.5 mmol/L (63 mg/dL). This higher threshold of blood glucose concentration is recommended in view of the absence of ketones as an alternative source of energy for the brain in this group of patients. Patients with HH have glucose requirements >8 mg/kg/min (normal glucose requirements: 4-6 mg/kg/min) and this is also one of the diagnostic criteria for HH (1).

The biochemical results will be informative only if taken at the time of hypoglycaemia. The diagnostic fast should be carried out in a controlled environment adjusting to the physiological fasting time expected for age. In HH, an inappropriate insulin and/or C-peptide concentration will be detected at the time of hypoglycaemia, with simultaneous poor response of ketone bodies and plasma non-esterified free fatty acids (1). The level of insulin achieved during hypoglycaemia is not indicative of the severity of the condition (1). A low insulin growth factor binding protein 1 (IGFBP-1) is also a marker of HH because the transcription of gene IGFBP-1 is suppressed by insulin (73). Other stress hormones such as cortisol and growth hormone should increase in the presence of hypoglycaemia and hence excluding these hormone deficiencies as the cause of hypoglycaemia. Urine organic acids, plasma amino acids, lactate and carnitines also need to be analysed in this test to rule out metabolic diseases. Table 1 summarises the biochemical criteria for HH.

In some cases, the diagnosis of HH can be difficult, so in these cases, certain stimulation tests can aid with the diagnosis. A positive glycaemic response (glucose increment of >1.5 mmol/L) to i.m./i.v. glucagon at the time of hypoglycaemia is compatible with HH (74). In HH, a positive glycaemic response will also be found after subcutaneous octreotide administration.

In HI/HA syndrome, the serum ammonia concentrations may be normal or raised (75), so the diagnosis requires a fast and a protein/leucine load test which will precipitate hypoglycaemia (30). Raised plasma hydroxyl-butyrylcarnitine and urinary 3-hydroxyglutarate are suggestive of HADH deficiency (36). If exercise-induced HH is suspected, then hypoglycaemia will need to be demonstrated in a formal exercise test or pyruvate load test (40). To confirm the diagnosis of postprandial HH, the test to use is a mixed meal test or an oral glucose tolerance test (76).

In those cases where there is no response to therapy with diazoxide, DNA on the patient and parents should be collected and sent for genetic analysis as the result will guide to the diagnosis (focal versus diffuse disease) and management. A genetic mutation will be found in 80-90% of diazoxide-unresponsive patients. Homozygous and compound heterozygous mutations in ABCC8 and KCNJ11 genes usually explain cases of diffuse disease, whereas paternally inherited mutations in these two genes are most commonly associated with focal disease (9). Mutations found in other genes not encoding the KATP channel will also shed light onto the appropriate management.

If the genetic findings are suggestive of focal disease, then the next step will be to perform 18F-DOPA PET-CT scan. The importance of this PET scan resides in the need to distinguish between the focal and the diffuse form of CHI as the surgical approach varies enormously between the two. This imaging technique has become, over the last years, the gold standard to localize the focal lesion within the pancreas (77) with the capacity to differentiate it from diffuse disease with 89% and 98% sensitivity and specificity, respectively (78). These results are much better than traditional imaging methods such as CT and MRI. Unlike trans-hepatic portal vein cannulation or pancreatic arteriography, PET scan is also appreciated for its non-invasiveness while maintaining high accuracy (79).

Pancreatic islets take up 18F-DOPA and convert it into dopamine using the enzyme DOPA decarboxylase. Focal and diffuse forms of HH have an increased activity of this enzyme (9). Uniform 18F-DOPA uptake throughout the whole pancreas is indicative of diffuse disease, in the contrary to the focal lesion, where the accumulation of 18F-DOPA within the lesion is markedly increased when compared to the surrounding tissue (80). The limitations of this technique are its availability in only a few centers around the world and the expertise required in the interpretation of the images.

## Management of the Different Forms of HH

The management of HH is very complex, yet a prompt and adequate therapy helps to prevent further episodes of hypoglycaemia, subsequent brain damage and other neurodevelopmental impairments (1).

The primary aim of management is to keep the blood glucose levels within the normal range (3.5-5.5 mmol/L) and to establish an appropriate fasting tolerance for age and a normal feeding pattern (15).

The management of HH may include dietary, medical and surgical approach, being in the majority of cases, a combination of these. The acute management is identical for all types of HH ensuring the stabilization of the blood glucose levels. This provides the clinician with time to determine the specific cause of HH and to introduce appropriate long-term therapy. Given the fact that it is a relatively rare condition with a challenging management, it is recommended that these patients are referred to tertiary centers that have the necessary experience and expertise in managing this condition (18).

### Acute Therapy

#### Parenteral glucose infusion:

The cornerstone of the immediate therapy is to provide sufficient glucose to maintain normoglycaemia. In respect to the biochemical basis of the hypoglycaemia (absence of ketone bodies), a higher threshold of blood glucose concentration should be aimed (>3.5 mmol/L) (81). The parenteral glucose requirements exceed 8 mg/kg/min and can often be as high as 15-25 mg/kg/min, thus in the severe forms of HH, the insertion of a central venous access may be required to deliver concentrated solutions of glucose (15,18,26). Parenteral glucose therapy can be supported with enteral feeding (15).

#### Frequent feeding:

 This is a very important supportive method in managing HH patients, although often difficult due to the feeding disturbances, food aversion, gastro-esophageal reflux disease and foregut dysmotility observed in patients with HH (82). These common problems can partially be explained as the side effects of the administered medicines, but also possibly influenced by an unknown mechanism shared by all types of HH patients. It is often necessary to deliver feeds via nasogastric tube or gastrostomy in order to keep blood glucose levels in the normal range.

#### Glucagon:

In case of emergency (e.g. symptomatic hypoglycaemia and seizures without a venous access), intramuscular administration of glucagon may be used (9,18,83,84). The recommended single dose is between 0.5-1 mg (82). Glucagon increases the blood glucose within a few minutes by inducing glycogenolysis, gluconeogenesis, ketogenesis and lipolysis (9,18,83,84). The use of glucagon has also been reported as a long-term management via a subcutaneous infusion (85). The dose should be 5-10 ug/kg/h if administered as an infusion (82). Glucagon can be used on its own or in a combination with octreotide in severe HH cases, when refractory hypoglycaemia persists despite high parenteral glucose intake. However, high doses of glucagon may paradoxically stimulate insulin secretion and increase glucose infusion requirements or cause further, rebound hypoglycaemia. Hence, glucagon doses above 10 ug/kg/h should be omitted (86).

### Long-Term Therapy

Further management of the different types of HH may differ. The identification of the specific type of HH can be done with help of rapid molecular genetic testing for the common CHI genes (ABCC8, KCNJ11) and targeted genetic testing for the less common genes if suggested by the phenotype. In indicated cases, further diagnosis can be made using 18F-DOPA PET-CT scan (9,15,18,79,83) to distinguish between the focal and the diffuse forms of CHI. In case of a focal form, the patients are cured by partial pancreatectomy, removing only the affected part of the pancreas that is producing excessive amount of insulin, while keeping the rest, ensuring sufficient exocrine and endocrine functions (84,87). On the other hand, in diffuse disease HH, the aim is to find a suitable medical therapy to avoid a near-total pancreatectomy (18,84).

The therapeutic approach will be different between those that are diazoxide-responsive and diazoxide-unresponsive cases. The management of diazoxide-responsive patients is straightforward, while the management of diazoxide-unresponsive cases is much more challenging. The clinician should try to find a suitable medical therapy or if necessary, in case of medically unresponsive cases, to resort to surgical therapy (9,18,82,83,88).

Identification of much rarer cases of CHI, such as GLUD1, HADH or SLC16A1 gene mutations, may transform management strategies in these patients. GLUD1 and HADH mutation-positive patients are usually protein-sensitive (29,37) and reducing the protein in the diet can significantly decrease the number of hypoglycaemic episodes that they experience. Whereas hypoglycaemia in SLC16A1 mutation carriers is exercise-induced and it is the anaerobic type of exercise that these patients need to eliminate in order to minimize the number of hypoglycaemic episodes (40).

**Figure 2:**
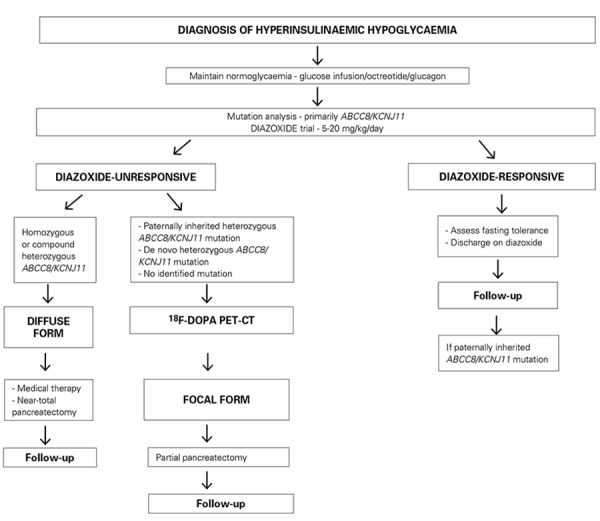
Proposed management algorithm for the treatment of congenital hyperinsulinism

A detailed management algorithm is summarized in Figure 2.

### Medical Therapy

The list of drugs used in the management of HH is summarized in Table 2.

#### Diazoxide:

The first-line therapy for all types of HH is the oral administration of diazoxide. Diazoxide binds to the SUR1 subunit causing opening of the intact KATP channels, which results in the blockade of β-cell depolarization and subsequent reduction of insulin secretion (1,9,18,19,83,89). This is basically the opposite mechanism of action than that observed with the oral hypoglycaemic agent sulphonylurea (90).

Based on the clinical response to therapy with diazoxide, the patients can be divided into two groups: diazoxide-responsive and diazoxide-unresponsive. In a recently published cohort of 300 patients, 63.5% were diazoxide-responsive and 36.5% diazoxide-unresponsive (5). This responsiveness is based on the fact that intact KATP channels are required for diazoxide to work. Therefore, children with diffuse disease due to inactivating mutations in ABCC8 and KCNJ11 and most patients with focal lesions are usually diazoxide-unresponsive (9,18,19,83,89). On the other hand, patients carrying mutations in the remaining known CHI genes are usually diazoxide-responsive (5,15).

To assess the effect of treatment with diazoxide, this should be started orally at 5 mg/kg/day in three divided doses and the dose can be gradually increased, if needed, up to a maximum dose of 20 mg/kg/day. Diazoxide should be tried on the maximum dose for at least 5 subsequent days before the patient can be described as unresponsive (89). For patients who do not respond to diazoxide, further increase of the dose would result in an increased risk of side effects.

If the patients are diazoxide-responsive, the positive effect on the blood glucose levels can often be observed before reaching the maximum dose, hence upper limit doses are not usually necessary. The most commonly observed and also the most serious side effect is fluid retention. It occurs mostly in the neonatal period and in at-risk patients may lead to the development of congestive heart failure and pulmonary hypertension (91). Therefore, chlorothiazide (5-10 mg/kg/day in 2 divided doses), a thiazide diuretic, is usually introduced together with diazoxide. It is used to prevent fluid retention, but it is also convenient for its synergistic effect on the suppression of insulin secretion (9,18,84). In older children, if there is no evidence of fluid retention, therapy with chlorothiazide is not necessary. Other common side effect of diazoxide is hypertrichosis, which may be very pronounced and could be a source of stress for the whole family. Fortunately, this side effect is fully reversible after discontinuation of therapy with diazoxide (91). Less common side effects may include nausea, vomiting, feeding problems, hyperuricaemia, tachycardia and leukopenia (91).

If the daily dose of diazoxide required to maintain normoglycaemia falls below 5 mg/kg/day, then discontinuation of the treatment should be considered in a hospital setting (88).

#### Octreotide:

This is the second line of medical therapy for children with diazoxide-unresponsive CHI. Octreotide is a long-acting somatostatin analogue that inhibits insulin secretion from pancreatic β-cells. This is mediated by binding to the somatostatin receptor SSTR5 which then inhibits calcium mobilization and acetylcholine activity therefore decreasing the insulin gene promoter activity which results in reduced insulin biosynthesis (90). Somatostatin may also exhibit an effect on insulin secretion through its action on the KATP channel (18).

The recommended dose of octreotide is 5-35 ug/kg/day and it can be administered either by 3-4 daily subcutaneous injections or as a continuous subcutaneous infusion (92). Octreotide exhibits a rapid increase of blood glucose level after administration of the first dose, but this may be followed by tachyphylaxis, which causes a rapid decline in response to octreotide 24-48 hours after initiation of therapy. Tachyphylaxis is generally transient and can be managed by dose adjustment (9,18,82,83). Potential adverse effects of octreotide include acute anorexia, nausea, abdominal pain, diarrhoea, drug-induced hepatitis, long QT syndrome and development of necrotizing enterocolitis (93). Long-term side effects include decreased intestinal motility, bile sludge, gallstone and suppression of pituitary hormones (growth hormone and thyroid stimulating hormone) (82).

#### Long-acting somatostatin analogues:

Recently, two prolonged-released formulations of synthetic somatostatin analogues - LAR-octreotide and lanreotide - have been successfully used in children with CHI (94). Long-acting octreotide is administered as intramuscular or deep subcutaneous injection every 4 weeks, which positively influences families’ compliance and improves the patients’ quality of life (82). Although the number of patients studied so far is limited and more studies are required to look into the long-term effectiveness of this medication, all the patients had the same or better response to long-acting octreotide than to previous medication (3-4 daily subcutaneous injections of short-acting octreotide and intensive feeding regime). Moreover, none of the severe side effects was observed (94).

#### Nifedipine:

Reduction of insulin secretion during administration of nifedipine has been described in several cases of patients with HH (95,96,97,98,99,100,101). Nifedipine is a calcium channel blocker and inhibits insulin secretion by inactivating the voltage-gated calcium channels. However, the vast majority of HH patients fails to show any response and calcium channel blockers are not regularly being used in the treatment of HH (96). But given the role of the voltage-gated calcium channels in regulating insulin secretion, more standardized studies and trials on HH patients are required before completely ruling out this medication from management of HH.

#### New Perspectives

##### Sirolimus:

Recently, the mammalian target of rapamycin (mTOR) inhibitor sirolimus has been successfully used in several patients with diffuse HH who were un-responsive to maximum doses of diazoxide and octreotide (102). The excessive activation of the mTOR pathway plays a role in the pathogenesis of HH (103). The use of mTOR inhibitor sirolimus reduces the β-cell proliferation and inhibits insulin production therefore resulting in a clinically significant glycemic response. The authors have not reported any major side effects during 1 year of follow-up (102).

##### Exendin:

The GLP-1 receptor antagonist exendin-(9-39) has recently been reported to elevate fasting blood glucose level in adults with KATP HH (104). Therefore, it has been suggested that exendin could represent a novel therapeutic target to manage hypoglycemia in HH patients. However, further clinical studies are needed to assess its effectiveness, safety, and pharmacokinetics.

##### Surgical Therapy

The indications for surgery in CHI patients include confirmed focal disease on 18F-DOPA-PET-CT scan and medically un-responsive diffuse disease.

##### Focal form:

The focal form of CHI has been reported in about 40-65% of all patients treated surgically (6,21). The treatment of choice for patients with the focal form of CHI is partial pancreatectomy. After removing the affected part of pancreas, the patients should be completely cured from the hypoglycaemia (105). The exact localization of the focal lesion is made using 18F-DOPA PET-CT that helps to guide the surgeon during the surgery (79). If the lesion is localized in the body or in the tail of the pancreas, laparoscopic approach should be used. Recent studies show that the accuracy and success of the laparoscopic approach is comparable to open surgery while benefiting from a shorter post-operative care and minor patients’ trauma after a keyhole surgery (106). On the other hand, if the lesion is in a surgically difficultly accessible location (for example in the pancreatic head), open laparotomy may be needed. In a group of 47 patients who underwent partial pancreatectomy for an evident focal lesion, 100% of cases were completely cured and none of them required insulin therapy after the operation (107).

##### Diffuse form:

The last resort for patients with medically unresponsive diffuse form of HH is a near-total pancreatectomy (21). However, some children remain hypoglycaemic despite the removal of 95-98% of pancreatic tissue. Then, a further attempt to control the condition with diazoxide therapy can be made. In a study that included 58 children who underwent near-total pancreatectomy, 59% of them remained hypoglycaemic immediately after surgery or later. However, the hypoglycaemic episodes were not as severe, occurred mainly pre-prandially, mostly at the end of an all-night fast and could be managed by adjusting the feeding regime or medication (107). In addition, with increasing age the severity of these hypoglycaemic episodes decreased and at the time of 5 years after surgery these episodes were recorded only in a few isolated cases (107). In a minority of cases, further re-operation and total pancreatectomy may be necessary to control severe CHI (87).

Near-total pancreatectomy unfortunately carries a high risk of exocrine pancreatic insufficiency and development of diabetes mellitus later in life (108). In some cases, hyperglycaemia may occur within the first days after surgery, thereafter, the incidence of hyperglycaemia gradually increases with age. The need for insulin therapy is 19% immediately after surgery and goes up to 91% at age of 14 years (107).

Given these risks, medical therapy is preferred when possible (87). In recent years, the number of patients undergoing these procedures has dropped, that is due to recent advances in pharmacotherapy.

##### Follow-up

Given the complexity of this condition, HH patients need to be regularly followed up in regards to their glycemic response but also in regards to their neurological development and other possibly related conditions in the case of syndromic HH. The parents/carers need to be educated in home blood glucose monitoring and what measures to take in case of hypoglycaemia (hypoplan).

Children with diffuse disease on medical therapy should have 24-hour blood glucose profile and fasting tolerance regularly checked to optimize medical therapy as they may need adjustments in the medication dose due to their weight gain. Some children, on the other hand, may need therapy adjustments as their HH gets milder as they grow older.

Children with diffuse disease that undergo subtotal (95%), near-total (98%) or subsequent total pancreatectomy have to be closely followed up particularly concentrating on signs for the onset of diabetes mellitus, as the percentage of children developing insulin-dependent diabetes following these procedures is extremely high (107).

In contrast, children with a focal form of CHI that have been completely cured following a resection of the pancreatic focal lesion do not require an intensive follow-up.

In case of CHI caused by genetic defects in pancreatic transcription factors genes (HNF4A, HNF1A), it is essential to adjust the dose of diazoxide as HH in these cases is transient and subsides in childhood. These children are then expected to develop non-autoimmune diabetes (MODY) later in early adulthood (46). It is therefore crucial that these children are not lost to follow-up and the nature of this genetic condition is properly explained to the parents as well as to the patients.

## Conclusion

HH is an important cause of hypoglycaemia in the newborn and childhood period, therefore prompt diagnosis is the key in the management of HH patients. Although there has been a huge progress in the diagnosis and management of patients with CHI, it still remains a challenging condition for the clinicians. Molecular genetics brings new possibilities into the diagnostics, unraveling the processes leading to hyperinsulinism. Along with novel imaging techniques (such as 18F-DOPA PET-CT), new medications and laparoscopic surgery have dramatically improved the outcome of CHI patients over the last decade. However, genetic etiology remains unknown in approximately 80% of diazoxide-responsive and 10% of diazoxide-unresponsive cases, thus suggesting that there are yet unidentified genetic causes of HH (109). Therefore, further research is required to identify new involved genes and to develop novel therapeutic therapies.

## Figures and Tables

**Table 1:**
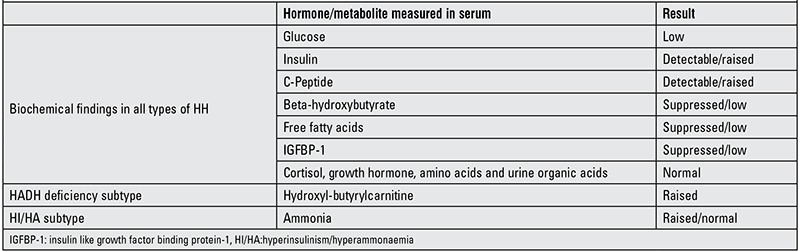
Biochemical findings in hyperinsulinemic hypoglycaemia (HH)

**Table 2:**
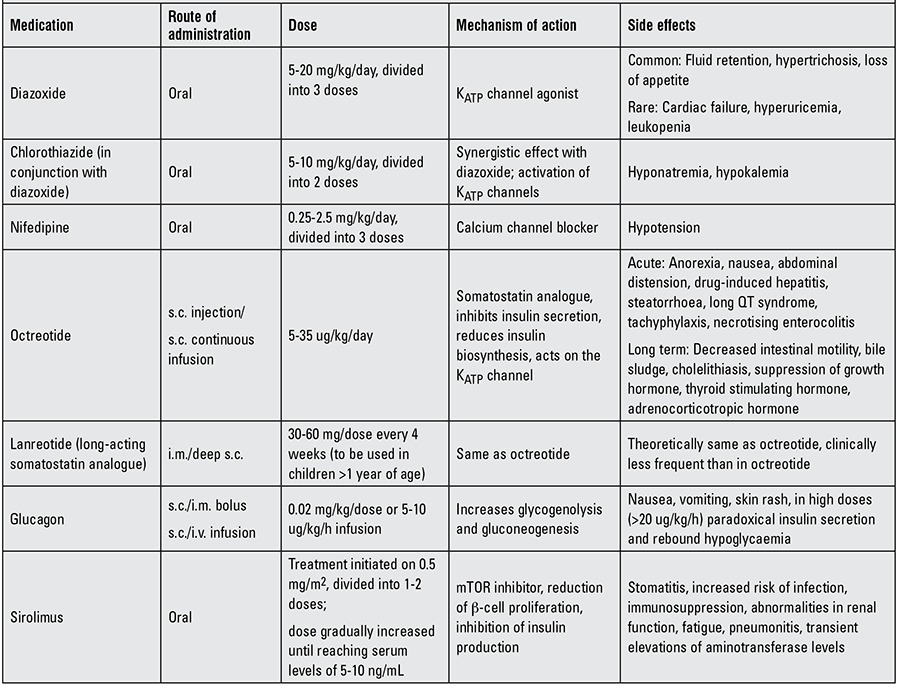
Drugs used in the management of congenital hyperinsulinemic hypoglycaemia
